# Public transit stop density is associated with walking for exercise among a national sample of older adults

**DOI:** 10.1186/s12877-023-04253-x

**Published:** 2023-09-26

**Authors:** Erica Twardzik, Jason R. Falvey, Philippa J. Clarke, Vicki A. Freedman, Jennifer A. Schrack

**Affiliations:** 1 Department of Epidemiology, Johns Hopkins Bloomberg School of Public Health, 2024 E Monument Street, Suite 2-700, Baltimore, MD 21205 USA; 2 Center on Aging and Health, Johns Hopkins University, Baltimore, MD USA; 3 Department of Physical Therapy and Rehabilitation Science, University of Maryland School of Medicine, Baltimore, MD USA; 4 Department of Epidemiology and Public Health, University of Maryland School of Medicine, Baltimore, MD USA; 5 Institute for Social Research, University of Michigan, Ann Arbor, MI USA; 6 Department of Epidemiology, School of Public Health, University of Michigan, Ann Arbor, MI USA

**Keywords:** Travel behavior, Geography, Exercise, Public transit; older adults

## Abstract

**Background:**

Walking is the primary and preferred mode of exercise for older adults. Walking to and from public transit stops may support older adults in achieving exercise goals. This study examined whether density of neighborhood public transit stops was associated with walking for exercise among older adults.

**Methods:**

2018 National Health and Aging Trends Study (NHATS) data were linked with the 2018 National Neighborhood Data Archive, which reported density of public transit stops (stops/mile^2^) within participants’ neighborhood, defined using census tract boundaries. Walking for exercise in the last month was self-reported. The extent to which self-reported public transit use mediated the relationship between density of neighborhood public transit stops and walking for exercise was examined. Covariates included sociodemographic characteristics, economic status, disability status, and neighborhood attributes. National estimates were calculated using NHATS analytic survey weights.

**Results:**

Among 4,836 respondents with complete data, 39.7% lived in a census tract with at least one neighborhood public transit stop and 8.5% were public transit users. The odds of walking for exercise were 32% higher (OR = 1.32; 95% confidence interval: 1.08, 1.61) among respondents living in a neighborhood with > 10 transit stops per mile compared to living in a neighborhood without any public transit stops documented. Self-reported public transit use mediated 24% of the association between density of neighborhood public transit stops and walking for exercise.

**Conclusions:**

Density of neighborhood public transit stops was associated with walking for exercise, with a substantial portion of the association mediated by self-reported public transit use. Increasing public transit stop availability within neighborhoods may contribute to active aging among older adults.

## Background

Public transit use is positively associated with physical activity within the general United States population [1–3]. Using public transit for everyday commuting facilitates routine-based physical activity. In a single public transit trip, a user will walk to a transit stop, potentially transfer to a connecting route(s), and walk from the final transit stop to an end destination. The first and last legs of a public transit trip make significant contributions to meeting physical activity recommendations [2], with a large proportion (29%) of transit users achieving 30 min of physical activity solely by walking to and from transit stops [1]. Overall, transit users spend a median of 20 min per day walking to and from transit stops, making it a sustainable source of physical activity [3]. Given the benefits of transit use for engagement in physical activity behavior, previous research has investigated if the accessibility of public transit is associated with public transit use and physical activity.

Evidence indicates that the built environment is an important contextual driver of individual public transit use and physical activity within the United States [4, 5]. The built environment comprises the physical environment that is directly created or modified by people [6]. Built environments contribute to the context in which people live and is an effective point for public health investigation given the broad reach of built environment interventions, sustainability of built environment modification, and reduced individual effort needed to shape behaviors over time [7]. Public transportation systems are a component of the built environment, and play a critical role in health and health behaviors of the population [8]. Previous research has found the density of neighborhood public transportation stops to be an important driver of individual public transit use in the United States [9]. Li and colleagues found that living in a neighborhood with high density of public transit stations was associated with more walking for transportation among adults in Portland, Oregon [9]. In addition, people living in areas with high density of public transit stations were more likely to meet physical activity recommendations [9]. However, studies using natural experiments to examine the relationship between the density of public transit stops and physical activity behavior in the United States have found mixed results [10]. Huang et al. found that installing 13 new light rail transit stations in Seattle, Washington resulted in increased transportation physical activity and decreased total physical activity among adults 18 and older [11]. In contrast, Miller et al. found that installing five new light rail transit stations in Salt Lake City, Utah resulted in increased transportation physical activity and total physical activity among adults 18 and older [12]. Additional research is needed to investigate if greater accessibility in public transportation stops is associated with walking behavior. Furthermore, the transferability of findings among the general United States population to older adults in the United States is not well understood.

The United States population is rapidly aging, making older adults an important public transportation user group. Public transportation is a key domain of urban life within the World Health Organizations framework for *Global Age-Friendly Cities* [13]. The accessibility of public transportation contributes to the process of active aging, defined as a process where opportunities are available for older adults to optimize their health, participation, and security as they age [13]. If public transit stops are available within the neighborhood, then older adults have greater opportunity to walk to public transit stops and maintain independent mobility. Alternatively, if there are no public transit stops within a neighborhood, older adults may have to rely on private transportation options (e.g., driving, family/friends) to maintain independent mobility. 20% of older adults do not drive, and most cease driving due to changes in capacity to drive a car because of age-related functional decline, disability, or both [14, 15]. Older adults with disabilities, who have an increased need for public transportation options, may face greater challenges accessing transit due to physical barriers in availability, accessibility, and delivery of public transportation services [16]. However, if public transportation is available and accessible to meet the needs of older adults with disabilities, it has the potential to enhance active aging.

To date, evidence of the relationship between density of neighborhood public transportation stops and walking behavior has been limited in geographic scope and has yet to investigate this relationship among older adults in the United States. The United States is a unique context to study public transportation impacts on walking behavior among older adults. The United States has long relied on automobiles as a primary form of individual transportation, however, with recent passing of the Infrastructure Investment and Jobs Act there may be a shift in transportation culture. The United States is investing $66 billion in passenger and freight rail and $39 billion in public transportation over the next five years to make public transportation more accessible [17]. Understanding relationships between the public transportation environment and older adults’ health behaviors is needed to inform future public transportation improvements. To date, the proportion of the relationship between neighborhood public transit stop density and physical activity mediated through individual public transit use among older adults has yet to be explored. Identification of relationships between neighborhood public transit density, individual public transit use, and individual walking behavior among older adults would provide foundational evidence to inform future physical activity promotion efforts among older adults through modification of the urban environment. Therefore, the primary aim of this study is to examine the relationship between the density of neighborhood public transportation stops and walking for exercise among older adults. It was hypothesized that greater density of fixed route transit stops within the neighborhood would be associated with greater likelihood to walk for exercise. As a secondary aim, this study investigates if the relationship between density of neighborhood public transit stops and walking for exercise is mediated by individual public transit use.

## Methods

### Data sources and study sample

This cross-sectional study uses data from the 2018 (round 8) wave of National Health and Aging Trends Study (NHATS). NHATS is a nationally representative sample of Medicare beneficiaries aged 65 and older living in the contiguous United States [18]. A stratified three-stage sampling design was used to construct the sampling frame, with counties or groups of counties as the primary sampling unit, ZIP codes or ZIP code fragments as the secondary sampling unit, and Medicare beneficiaries as the third sampling unit. Oldest age groups and Black non-Hispanic Medicare beneficiaries were oversampled. In 2018, the weighted response rate of participants was 94.0% [19]. Additional details on study design have been previously published [20]. Data collection has occurred on an annual basis since 2011, with detailed information collected through in-home interviews about participants’ health, well-being, and surrounding environments. The majority of NHATS participants responded to interview questions for themselves. However, 12.0% of participants during the 2018 NHATS interview could not respond, and information was collected through proxy report. The current study makes use of NHATS data collected in 2018, representing adults aged 68 and older, to align with the primary exposure data which was captured in 2018. Participants provided written informed consent to be a part of NHATS, and this study was approved by a local Institutional Review Board.

### Primary exposure

Density of neighborhood public transit stops within participant’s census tract was obtained from the National Neighborhood Data Archive [21]. The National Neighborhood Data Archive calculates density of neighborhood public transit stops within each census tract in the United States using data from the National Transit Map (NTM). NTM compiles General Transit Feed Specification data provided by 270 regional transit authorities in the United States and is continually updated by participating agencies [22]. The 270 participating regional transit authorities represented in the NTM dataset includes static information on locations where fixed-guideway and fixed-route pick up and drop off riders (i.e., transit stops). Stops within the NTM dataset include various modes of public transport (e.g., bus, subway, rail) available. Number of neighborhood transit stops in 2018 were captured and aggregated by researchers in April 2019 [21]. Density of neighborhood transit stops were calculated per square mile within 2010 census tract boundaries. Density of neighborhood transit stops was categorized into three groups: no transit stops documented, at least one transit stop and less than 10 transit stops per square mile, and greater than 10 transit stops per square mile.

#### Primary outcome

Walking for exercise was measured during the annual interview using self-report by asking participants whether they ever walked for exercise in the last month. A binary response of ‘Yes’ or ‘No’ was recorded.

#### Mediator

Individual public transit use was self-reported by participants during the annual interview. Participants were asked “In the last month, how did you get to places outside your home? Did you take public transportation (the bus, subway, or train)?” Participants provided a binary response of ‘Yes’ or ‘No’.

### Covariates

Several variables associated with physical activity [23] and access to transit [24–26] were included as confounders within our analysis. According to travel behavior theory, the major determinants of travel patterns are social class position, ethnicity, life cycle status, and residential location [27]. Therefore, potential confounding variables included sociodemographic characteristics (i.e., age, gender, race/ethnicity, marital status, number of people in social network), economic status (i.e., education, home ownership), disability status, and neighborhood attributes (i.e., geographic residence, duration of residence, neighborhood physical disorder, and social cohesion). Age was categorized into 5-year age brackets representing participants aged 68–69, 70–74, 75–79, 80–84, 85–89, and 90 or greater. Gender was self-reported as a binary variable for males and females. Race and ethnicity were self-reported and categorized into four groups including non-Hispanic White, non-Hispanic Black, non-Hispanic other, and Hispanic. Marital status was self-reported by participants and categorized into six groups representing married, living with a partner, separated, divorced, widowed, and never married. Number of people in social network was calculated from responses to a request to name the people the older adult talked with most often in the last year about important things. Up to five social network members could be named.

For economic status, participants were asked what the highest degree or level of school they completed. Education was then categorized into three groups representing less than high school, high school graduate, or more than high school. Homeownership, a measure of wealth, was self-reported during the interview and categorized as ‘Yes’ (i.e., own their home) or ‘No’ (i.e., rent their home, some other arrangement to live in their home).

Disability was captured via self-report and includes six indicator variables reflecting standard disability domains [28]: (1) visual impairment included reported blindness, difficulty seeing across the street even while wearing glasses, or difficulty reading newspaper print while wearing glasses [18]; (2) hearing impairment included reported deafness, use of a hearing aid or other hearing device, difficulty carrying a conversation with background noise, or inability to hear well enough to use a telephone [18];  (3) cognitive impairment included proxy or self-report rating that their memory was fair, or poor [18]; (4) mobility impairment included reported inability to walk 3 blocks or up 10 stairs [18, 29]; (5) self-care impairment included difficulty by oneself or never doing by oneself the following activities: eating, bathing, toileting, or dressing [18, 30]; and (6) communication impairment included reported difficulty speaking or making themselves understood while talking.

Four neighborhood attributes were treated as covariates within this study: (1) geographic residence was categorized as metropolitan or non-metropolitan county derived from Rural-Urban Continuum Codes [31, 32]; (2) duration of residence was categorized as living in their current location of less than five years or five year or more; (3) Neighborhood physical disorder was recorded by NHATS interviewers, where they recorded the extent of physical disorder (e.g., litter, graffiti, vacant houses, and continuous sidewalks) surrounding the participant’s home on a four-point scale and reduced to a binary variable representing any neighborhood physical disorder or no neighborhood physical disorder; and (4) NHATS respondents self-reported their perception of community (i.e., how well people know each other, if people are willing to help each other, and if people in the community can be trusted) on a three-point scale [33, 34] and social cohesion was categorized into tertiles.

### Analytic strategy

This study focused on older adults living in the community or residential care settings other than nursing homes in 2018 to examine the association between density of neighborhood public transportation stops and walking for exercise. National estimates of density of neighborhood public transportation stops, disability status, and walking for exercise were obtained using analytic survey weights. The NHATS analytic survey weight accounts for differential selection probabilities and adjusts for nonresponse bias [20]. Our analysis was restricted to participants living in the community or residential care settings other than nursing homes who had non-missing data. Using the survey command suite within STATA, logistic regression was performed to assess associations between density of public transportation stops and walking for exercise. Using a sequential model building strategy, associations between density of public transit stops and odds of walking for exercise in the last month were assessed. Unadjusted estimates are evaluated in Model 1, Model 2 adjusts for demographic characteritiscs, Model 3 additionally adjusts for economic characteristics, Model 4 additionally adjusts for disability status, and Model 5 adjusts for all theorized covariates.

**Fig. 1:**
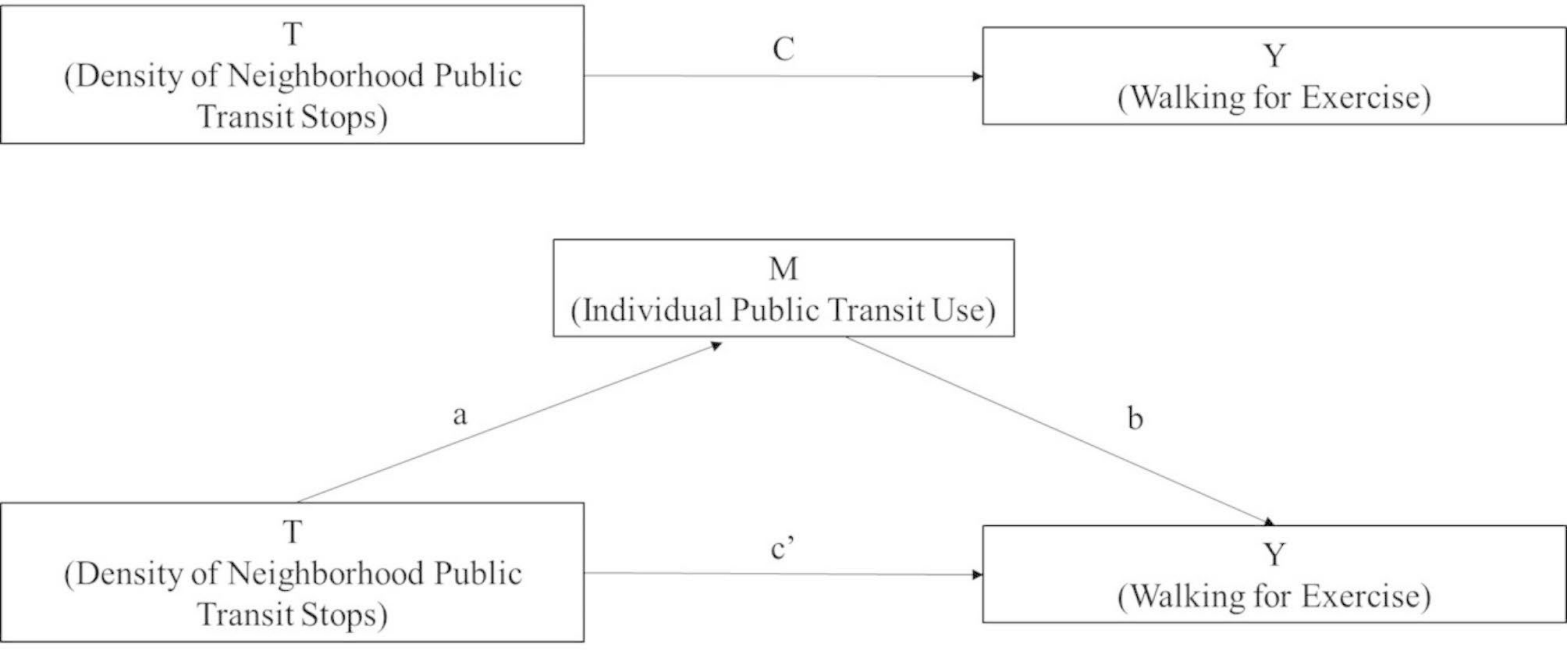
Hypothesized direct and indirect pathways linking density of public transit stops to walking for exercise through public transit use among National Health and Aging Trends Study respondents living in the community or residential care settings other than nursing homes, United States, 2018

The extent to which individual public transit use mediated the association between density of neighborhood public transit stops and walking for exercise was assessed using the *STATA medeff* package for causal mediation analysis [35–37]. This study hypothesized that the relationship between density of neighborhood public transit stops and walking for exercise would be mediated through individual public transit use. However, there may be other mechanisms through which density of neighborhood public transit stops influence walking for exercise among older adults. For example, density of neighborhood public transit stops is correlated with other features of the neighborhood context known to be assoicated with physical activity, such as median household income [38] and land use [39]. Figure 1 displays the hypothesized causal mechanism through individual public transit use. The mediator was modeled with a logistic regression, using the same sequential model building strategy as described above. The outcome model was a logistic regression including the mediator and sequential model building strategy as described above. Average causal mediation effect was computed by taking the difference between the estimated total effect (Fig. 1, path C) and the average direct effect (Fig. 1, path c’). All analyses were conducted using STATA 16.1.

## Results

The 2018 round of NHATS collected data on 5,547 respondents. Respondents were excluded from the current study if they had died (n = 397), lived in a nursing home at the time of the interview (n = 232), were not administered an interview (n = 81), or had missing item-level information (n = 1). A total of 711 were excluded, resulting in a final analytic sample of 4,836.

As shown in Table 1, most participants (60.3%) lived in a neighborhood with no documented public transportation stops available, followed by 23.2% living in a neighborhood with more than 10 public transit stops per square mile and 16.5% living in a neighborhood with 0–10 public transit stops per square mile. Many participants reported walking for exercise in the last month (62.3%) and few participants used public transit (8.5%). Compared to the total study sample, a greater proportion of participants living within neighborhoods with high density of public transit stops self-identified as Black non-Hispanic (16.2% vs. 7.9%), Hispanic (11.1% vs. 7.4%), and separated/divorced (20.8% vs. 14.1%). In addition, a greater proportion of neighborhoods with high density of public transit stops had observed physical disorder (11.5% vs. 7.8%) and low levels of social cohesion (16.8% vs. 13.0%) compared to the total study sample. The proportion of participants who reported individual public transit use was differential by density of neighborhood public transit stops, ranging from 4.8% of participants using public transit among those living in a neighborhood with 0–10 public transit stops per square mile to 19.5% of participants using public transit among those living in a neighborhood with greater than 10 public transit stops per square mile. Additional details on descriptive statistics of individual and environmental characteristics can be found in Table 1.

**Table 1:** Characteristics of older adults living in the community or residential care settings other than nursing homes within the 2018 National Health and Aging Trends Study (NHATS) survey, stratified by the density of public transit stops within participant’s census tract

	Density of transit stops (stops/mi^2^)							
	X = 0		0 < X < = 10		X > 10		Total	
Sample characteristic^a^	(n = 2,915)		(n = 798)		(n = 1,123)		(n = 4,836)	
Ever go walking for exercise								
Yes	1,287	40.3%	314	33.9%	455	33.3%	2,056	37.7%
No	1,628	59.7%	484	66.1%	668	66.7%	2,780	62.3%
Type of Respondent								
Self-report	2,804	97.4%	756	96.3%	1,058	96.2%	4,618	96.9%
Proxy	111	2.7%	42	3.7%	65	3.8%	218	3.1%
Age								
68 to 69	116	8.8%	26	7.5%	49	10.6%	191	9.0%
70 to 74	685	37.2%	183	38.5%	222	31.9%	1,090	36.3%
75 to 79	723	23.9%	196	24.1%	297	25.9%	1,216	24.3%
80 to 84	623	15.7%	157	13.8%	238	16.0%	1,018	15.4%
85 to 89	454	9.1%	139	10.0%	175	9.2%	768	9.3%
90+	314	5.4%	97	6.2%	142	6.4%	553	5.7%
Gender								
Male	1,239	44.3%	327	44.8%	462	44.7%	2,028	44.5%
Female	1,676	55.7%	471	55.2%	661	55.3%	2,808	55.5%
Race and Ethnicity								
White Non-Hispanic	2,233	82.6%	594	79.4%	552	64.2%	3,379	78.2%
Black Non-Hispanic	438	5.7%	121	5.9%	428	16.2%	987	7.9%
Other	93	5.4%	36	7.8%	62	8.5%	191	6.5%
Hispanic	151	6.3%	47	6.9%	81	11.1%	279	7.4%
Marital Status								
Married	1,397	54.7%	358	52.0%	397	43.4%	2,152	51.9%
Living with a partner	54	2.5%	18	2.6%	22	2.0%	94	2.4%
Separated/divorced	332	11.9%	105	13.5%	225	20.8%	662	14.1%
Widowed	1,040	27.9%	289	28.6%	413	28.5%	1,742	28.2%
Never married	92	2.9%	28	3.3%	66	5.3%	186	3.5%
Number of people in social network, mean (SD)	2.2	(1.4)	2.3	(1.4)	2.1	(1.4)	2.2	(1.4)
Education								
Less than high school	824	27.4%	179	21.2%	224	17.9%	1,227	24.3%
High school	606	17.2%	121	13.7%	274	19.4%	1,001	17.1%
More than high school	1,485	55.3%	498	65.1%	625	62.8%	2,608	58.6%
Home ownership								
Yes	1,561	52.2%	351	40.7%	407	35.7%	2,319	46.8%
No	1,354	57.8%	447	59.3%	716	64.3%	2,517	53.2%
Vision impairment								
Yes	293	7.8%	82	9.1%	128	10.6%	503	8.6%
No	2,622	92.2%	716	90.9%	995	89.4%	4,333	91.4%
Hearing impairment								
Yes	881	27.8%	230	24.1%	268	21.4%	1,379	25.8%
No	2,034	72.2%	568	75.9%	855	78.6%	3,457	74.2%
Mobility impairment								
Yes	1,094	29.2%	293	27.8%	435	28.5%	1,822	28.8%
No	1,821	70.8%	505	72.2%	688	71.5%	3,014	71.2%
Cognitive impairment								
Yes	824	24.2%	191	19.0%	337	24.8%	1,352	23.4%
No	2,091	75.8%	607	81.0%	786	75.2%	3,484	76.6%
Self-Care impairment								
Yes	814	23.0%	222	22.9%	327	23.2%	1,363	23.0%
No	2,101	77.1%	576	77.1%	796	76.8%	3,473	77.0%
Communication impairment								
Yes	217	6.5%	65	7.1%	92	6.9%	374	6.7%
No	2,698	93.5%	733	92.9%	1,031	93.1%	4,462	93.3%
Metro area^b^								
Metro							3,913	82.3%
Nonmetro							923	17.8%
Residential duration								
< 5 years	492	17.6%	185	23.7%	201	17.0%	878	18.6%
>= 5 years	2,423	82.4%	613	76.3%	922	83.0%	3,958	81.4%
Neighborhood physical disorder								
None	2,611	89.8%	749	94.4%	948	86.2%	4,308	89.9%
Any	250	8.0%	28	3.0%	146	11.5%	424	7.8%
Missing	54	2.2%	21	2.6%	29	2.3%	104	2.3%
Social cohesion								
Agree a lot	967	32.2%	231	27.9%	280	25.9%	1,478	30.1%
Agree a little	1,431	50.1%	397	52.7%	543	48.7%	2,371	50.3%
Do not agree	338	11.9%	108	12.3%	181	16.8%	627	13.0%
Missing	179	5.9%	62	7.1%	119	8.7%	360	6.7%
Public Transit Use								
Yes	147	5.9%	35	4.8%	193	19.5%	375	8.5%
No	2,768	94.2%	763	95.2%	930	80.5%	4,461	91.5%

^a^Reported as n (weighted %) unless otherwise specified

^b^Summary of metro area stratified by public transit stop density suppressed due to small sample size

Abbreviations: SD, standard deviation

Table 2 presents the sequentially adjusted odds ratios (OR) and 95% confidence intervals (CI) for walking for exercise. Within the unadjusted model (model 1) the odds of walking for exercise among participants living in a neighborhood with 0–10 transit stops per square mile was 1.32 (95% CI: 1.07, 1.63) times the odds of walking for exercise among participants living in a neighborhood with no transit stops. Similar effect estimates were observed among participants living in a neighborhood with more than 10 transit stops per square mile (OR = 1.36; 95% CI: 1.10, 1.67). Associations were attenuated after adjustment for demographic, economic, impairment, and neighborhood characteristics. Within the fully adjusted model, the odds of walking for exercise did not significantly differ between participants living in a neighborhood with 0–10 transit stops per square mile compared to participants living in a neighborhood with no transit stops (OR = 1.20; 95% CI: 0.96, 1.49). Odds of walking for exercise among participants living in a neighborhood with more than 10 transit stops per square mile was 1.32 (95% CI: 1.08, 1.61) times the odds of walking for exercise among participants living in a neighborhood with no transit stops. In addition to the density of public transit stops in the neighborhood, several sociodemographic variables significantly contributed to the likelihood of walking for exercise. Participants who self-identified as Hispanic (OR = 1.61) or Other (OR = 1.66) race and ethnicity compared to non-Hispanic White and greater educational attainment (OR = 1.23) had significantly higher odds of walking for exercise. One additional person within a participant’s social network was associated with 15% higher odds (OR = 1.15; 95% CI: 1.09, 1.22) of walking for exercise. Lastly, mobility impairment (OR = 0.27), longer residential duration (OR = 0.68), and lower social cohesion (OR = 0.73 & OR = 0.65) were all significantly associated with lower likelihood of walking for exercise.

**Table 2:** Results from sequentially adjusted logistic regression analysis examining the association between number of public transit stops per square mile within a participant’s census tract and self-reported ever walking for exercise in the last month (n = 4,836)

	Model 1		Model 2		Model 3		Model 4		Model 5	
	OR	95% CI	OR	95% CI	OR	95% CI	OR	95% CI	OR	95% CI
Density of transit stops (stops/mi^2^)										
X = 0	ref		ref		ref		ref		ref	
0 < X ≤ 10	1.32*	(1.07, 1.63)	1.30*	(1.05, 1.60)	1.25*	(1.01, 1.54)	1.24*	(1.00, 1.52)	1.20	(0.96, 1.49)
X > 10	1.36**	(1.10, 1.67)	1.43**	(1.16, 1.76)	1.36**	(1.11, 1.67)	1.31**	(1.07, 1.60)	1.32**	(1.08, 1.61)
Type of Respondent										
Self-report			ref		ref		ref		ref	
Proxy			0.61*	(0.41, 0.92)	0.64*	(0.42, 0.95)	1.13	(0.74, 1.73)	1.04	(0.68, 1.59)
Age										
68 to 69			ref		ref		ref		ref	
70 to 74			0.98	(0.73, 1.32)	0.98	(0.73, 1.32)	0.95	(0.71, 1.27)	0.95	(0.71, 1.28)
75 to 79			0.74*	(0.56, 0.98)	0.75	(0.57, 1.00)	0.81	(0.62, 1.06)	0.82	(0.63, 1.06)
80 to 84			0.60**	(0.45, 0.81)	0.63**	(0.47, 0.84)	0.77	(0.58, 1.03)	0.76	(0.58, 1.00)
85 to 89			0.55***	(0.40, 0.74)	0.57***	(0.42, 0.77)	0.84	(0.63, 1.11)	0.83	(0.64, 1.09)
90+			0.47***	(0.35, 0.64)	0.50***	(0.37, 0.67)	0.94	(0.68, 1.32)	0.93	(0.67, 1.28)
Gender										
Male			ref		ref		ref		ref	
Female			0.78**	(0.66, 0.91)	0.79**	(0.68, 0.93)	0.88	(0.75, 1.02)	0.89	(0.76, 1.04)
Race and Ethnicity										
White Non-Hispanic			ref		ref		ref		ref	
Black Non-Hispanic			0.76**	(0.62, 0.92)	0.83	(0.68, 1.02)	0.94	(0.74, 1.20)	0.99	(0.78, 1.25)
Other			1.36	(0.90, 2.05)	1.53*	(1.01, 2.34)	1.63*	(1.02, 2.58)	1.66*	(1.03, 2.69)
Hispanic			1.19	(0.86, 1.66)	1.41	(1.00, 1.99)	1.57**	(1.14, 2.14)	1.61**	(1.15, 2.26)
Marital Status										
Married			ref		ref		ref		ref	
Living with a partner			1.08	(0.71, 1.65)	1.15	(0.75, 1.77)	1.21	(0.76, 1.93)	1.27	(0.81, 2.00)
Separated/divorced			1.06	(0.84, 1.33)	1.09	(0.86, 1.38)	1.17	(0.90, 1.52)	1.19	(0.91, 1.54)
Widowed			0.88	(0.72, 1.07)	0.91	(0.75, 1.11)	0.99	(0.80, 1.23)	0.98	(0.78, 1.21)
Never married			0.63*	(0.41, 0.97)	0.65	(0.42, 1.03)	0.75	(0.47, 1.19)	0.76	(0.48, 1.21)
Number of People in Social Network			1.20***	(1.13, 1.27)	1.18***	(1.11, 1.25)	1.17***	(1.10, 1.24)	1.15***	(1.09, 1.22)
Education										
Less than high school					0.93	(0.73, 1.18)	0.99	(0.78, 1.25)	0.99	(0.77, 1.26)
High school					ref		ref		ref	
More than high school					1.43***	(1.20, 1.71)	1.28*	(1.06, 1.54)	1.23*	(1.03, 1.48)
Home ownership										
No					ref		ref		ref	
Yes					1.01	(0.85, 1.20)	0.89	(0.74, 1.06)	0.92	(0.77, 1.11)
Vision impairment										
No							ref		ref	
Yes							0.90	(0.68, 1.20)	0.91	(0.69, 1.21)
Hearing impairment										
No							ref		ref	
Yes							1.00	(0.86, 1.16)	0.99	(0.84, 1.15)
Mobility impairment										
No							ref		ref	
Yes							0.27***	(0.22, 0.32)	0.27***	(0.22, 0.32)
Cognitive impairment										
No							ref		ref	
Yes							0.90	(0.76, 1.06)	0.92	(0.77, 1.10)
Self-Care impairment										
No							ref		ref	
Yes							0.88	(0.71, 1.10)	0.87	(0.71, 1.07)
Communication impairment										
No							ref		ref	
Yes							0.92	(0.67, 1.27)	0.91	(0.65, 1.26)
Metro area										
Metro									ref	
Nonmetro									0.84	(0.63, 1.12)
Residential duration										
< 5 years									ref	
>= 5 years									0.68**	(0.53, 0.86)
Neighborhood physical disorder										
None									ref	
Any									0.95	(0.69, 1.30)
Missing									0.83	(0.52, 1.34)
Social cohesion										
Agree a lot									ref	
Agree a little									0.73**	(0.60, 0.90)
Do not agree									0.65**	(0.49, 0.85)
Missing									0.41***	(0.31, 0.54)

Abbreviations: OR = Odds ratio; CI = confidence interval; NHATS = National Health and Aging Trends Study

* p < 0.05, ** p < 0.01, *** p < 0.001

*Note.* Model 1: unadjusted. Model 2: adjusted for age, sex, race/ethnicity, marital status, and number of people in social network. Model 3: model 2 + education and homeownership. Model 4: model 3 + vision impairment, hearing impairment, mobility impairment, cognitive impairment, self-care impairment, and communication impairment. Model 5: model 4 + metropolitan status, residential duration, neighborhood physical disorder, and social cohesion

Table 3 presents our sequentially adjusted mediation analysis. Within unadjusted models, individual public transit use mediated 46.6% of the association between density of neighborhood public transit stops and walking for exercise. After adjustment for all covariates the proportion of association mediated by individual public transit use decreased to 23.5%.

**Table 3:** Results from sequentially adjusted mediation analysis examining the extent to which public transit use mediates the association between number of public transit stops per square mile within a participant’s census tract and self-reported ever walking for exercise in the last month (n = 4,836)

	ACME Mean (95% CI)	ADE Mean (95% CI)	Total Effect Mean (95% CI)	% Total Effect Mediated Mean (95% CI)
Model 1	0.008 (0.006, 0.011)	0.009 (-0.008, 0.026)	0.017 (0.001, 0.035)	0.466 (0.196, 3.013)
Model 2	0.006 (0.004, 0.009)	0.026 (0.010, 0.042)	0.032 (0.016, 0.049)	0.200 (0.131, 0.406)
Model 3	0.005 (0.004, 0.007)	0.019 (0.001, 0.036)	0.024 (0.125, 0.664)	0.217 (0.127, 0.673)
Model 4	0.004 (0.003, 0.006)	0.014 (-0.003, 0.031)	0.018 (0.001, 0.035)	0.239 (0.107, 1.260)
Model 5	0.005 (0.003, 0.007)	0.016 (-0.001, 0.033)	0.021 (0.004, 0.038)	0.235 (0.128, 0.998)

Abbreviations: CI = confidence interval; ACME = average causal mediation effect; ADE = average direct effect

Model 1: unadjusted. Model 2: adjusted for age, sex, race/ethnicity, marital status, and number of people in social network. Model 3: model 2 + education and homeownership. Model 4: model 3 + vision impairment, hearing impairment, mobility impairment, cognitive impairment, self-care impairment, and communication impairment. Model 5: model 4 + metropolitan status, residential duration, neighborhood physical disorder, and social cohesion

## Discussion

In a nationally representative cohort study of older adults, this study found that living in an area with a high density of neighborhood public transit stops (i.e., more than 10 transit stops per square mile) was associated with greater odds of walking for exercise. The observed relationship was significant after accounting for sociodemographic characteristics, economic status, disability status, and neighborhood characteristics. Findings suggest that living in a neighborhood with better access to public transit service might shape individual public transit use and facilitate walking behavior. On a population health level, these findings have significant public health implications that point towards public transportation systems and urban development strategies as potential approaches to promote physical activity among older adults. Public transportation agencies can increase the number of stops within residential area to make transit more accessible to older adults.

Additionally, this study explored the extent to which individual public transit use mediates the association between density of neighborhood public transit stops and walking for exercise. This study found that individual public transit use mediated 24% of the relationship between density of neighborhood public transit stops and walking for exercise, indicating that greater availability of public transit stops within neighborhoods (i.e., density of public transit stops) is associated with higher individual public transit use and higher individual public transit use is associated with walking for exercise. Walking for exercise is an important health goal for older adults that is associated with reductions in mortality, cardiovascular disease, type 2 diabetes, musculoskeletal disorders, cancer, and obesity [40]. Furthermore, physical activity has important benefits to older adults’ quality of life through improvements in sleep, cognitive function, and mental health [40]. The remaining 76% of the relationship was not mediated through individual public transit use, suggesting there are other mechanisms through which density of neighborhood public transit stops is associated with walking for exercise among older adults. One potential theory is that areas with greater density of public transit stops may have other features of the built environment, such as diversity of land use, intersection density, and number of destinations, which have been shown to be strongly related to walking behavior [41]. For example, areas with greater density of public transit stops could also have greater access to retail establishments (e.g., grocery stores, shopping malls) or destinations for social engagement (e.g., coffee shops, places of worship) to which older adults may be motivated to walk [41]. Additional research is needed to investigate features of the built environment and behavioral components that either facilitate or hinder public transit access and walking for exercise among older adults.

The findings from the current study align with previous work investigating the association between public transit use and physical activity behavior in the general population [42–44]. Among a group of adults in King County, Washington, public transit use was associated with greater physical activity and walking behavior compared to no public transit use [42]. Furthermore, this relationship was greatest in magnitude among the most frequent transit users [42]. Similarly, public transit use was associated with greater physical activity behavior in Atlanta [43], New York City [44], and across North America [1]. Within the English Longitudinal Study of Ageing, research has demonstrated that access to a bus pass among older adults makes transportation more accessible and thereby associated with greater physical activity within this population subgroup [45, 46]. The current study adds to this body of literature by estimating relationships among a nationally representative sample of United States older adults, while accounting for disability status. This study found that density of neighborhood public transit stops in a census tract and individual public transit use were associated with walking for exercise among older adults, above and beyond disability. Taken together, our findings suggest that increasing density of public transit stops, and thereby facilitating greater individual public transit use, is one strategy to improve physical activity participation among older adults. Using catalytic forecasting to quantify public transit demand based on population demographics, including the composition of older adults within a community, is a promising strategy to improve access and equity in public transportation [47]. Older adults should be a priority population for public transit equity given the physical activity promotion benefits of public transit use and large proportion of non-driving older adults in the United States [48, 49].

Beyond facilitating improvements in walking for exercise among older adults, improving public transit infrastructure and facilitating access to transit has additional benefits for older adults [50, 51]. Older adults are at greater risk of transportation disadvantage compared to younger adults [52], and transportation is a common concern to accessing health care among older adults. Over 16% of older adults report transportation barriers to healthcare, and have missed care because of a problems with transportation in the United States [53]. Improving the density and accessibility of neighborhood public transit may mitigate the risks of transportation disadvantage among older adults and could provide greater access to the health care system. However, modifying and adapting the built environment to meet the needs of older adults will take time. Therefore, while addressing the physical barriers to public transit access there are other interventions (e.g., fare vouchers, travel training programs) that can be put into place to expand access and use of public transit among older adults. Public transit offers older adults’ greater autonomy, independence, and quality of life. Reduced or restricted transportation access has been associated with social isolation, depression, and mortality among older adults [54, 55]. As demonstrated by the results of the mediation analysis, individual public transit use promotes walking for exercise, making public transit use a key component of active aging.

This study has several strengths. We draw upon a novel national database objectively identifying neighborhood public transportation stops. The point locations of public transit stops were aggregated to the census tract level and linked with NHATS participants’ home addresses. In addition, this study adds to the current body of evidence by demonstrating the role that density of neighborhood public transportation stops has on walking for exercise among older adults. This is the first study to our knowledge that has examined the association between density of public transportation stops and walking for exercise among a geographically diverse, nationally representative sample of older adults. In addition, this study integrates rich detail on disability status, neighborhood physical disorder, and neighborhood social cohesion within our models providing robust effect estimates of the relationship between density of public transit use and walking for exercise. Furthermore, using a nationally representative sample of older adults brings greater external validity to the observed associations within this study.

However, this study is not without limitations. The study findings are limited in external validity. These results are generalizable to adults 68 years or older living in the community or residential care settings other than nursing homes. Additionally, due to voluntary participation in NTM, a value of 0 may indicate either an absence of transit stops within a census tract, or the non-participation of a regional transit authority in NTM [21]. Since values of 0 have different meanings, this introduces information bias. Specifically, differential misclassification of our primary exposure can bias our effect estimates. We expect that misclassification of census tracts to a value of 0 due to non-participation of regional transit authorities in the NTM would bias estimates towards the null. This means that the effect estimates potentially underestimate the true association between public transit stop density on walking for exercise among older adults. Although NTM participation was voluntary, it includes data from over 270 transit agencies, providing information on over 398,000 stops and stations along 10,000 routes within the United States [22]. Furthermore, our research is limited by the quantity of neighborhood public transit stops and were unable to collect information about the quality of neighborhood public transportation stops (e.g., shelter, bench, lighting), which may serve as a major facilitator for older adults’ use of the public transit system. Participants also self-reported if they walked for exercise in the last month, a crude estimate for physical activity participation [56]. The binary measurement of metro area used in this research does not fully capture the heterogeneity in the rural-urban continuum. Previous research has shown that the relationship between environmental features and physical activity varies by urbanicity [57]. Additional research is needed to investigate effect measure modification by urbanicity with great representation of the heterogeneity among non-urban participants. There is the potential that unmeasured confounders, such as climate and weather, may be present and distort the true underlying relationship between transit stop density and walking for exercise among older adults. Lastly, there are many components of the travel chain that were not captured within this project, including the walkability of the neighborhood environment (e.g., residential density, street connectivity, and land use mix). Previous research has shown that neighborhood walkability is associated with greater likelihood of individual transit use, and future research should take these attributes into consideration [58].

## Conclusions

Within a nationally representative sample of older adults within the United States, this study found that the density of neighborhood public transit stops was associated with walking for exercise in the last month. A substantial portion of this association (24%) mediated through self-reported individual public transit use. Increasing the availability of public transit within neighborhood environments may contribute to active aging among older adults and facilitate aging in place within the United States. Therefore, increasing the density and availability of public transit stops may be a modifiable intervention target to promote public transit use and walking for exercise among older adults.

## Data Availability

The datasets generated and/or analyzed during the current study are available in the NHATS and NaNDA repositories, www.nhats.org and https://nanda.isr.umich.edu/data/.

## Abbreviations

NHATS: National Health and Aging Trends Study
NTM: National Transit Map
